# A gene-expression signature defines a subtype of Stomach Adenocarcinomas with low levels of Claudins and a high ratio of NF-YA long/NF-YA short splicing variants

**DOI:** 10.1007/s10120-025-01671-1

**Published:** 2025-10-13

**Authors:** Alberto Gallo, Mirko Ronzio, Maria Barbara Campbell, Sofia Polettini, Enrico Garattini, Roberto Mantovani, Diletta Dolfini

**Affiliations:** 1 Dipartimento di Bioscienze, Università degli Studi di Milano, Via Celoria 26, 20133 Milan, Italy; 2 Dipartimento di Biochimica e Farmacologia Molecolare, Istituto di Ricerche Farmacologiche Mario Negri IRCCS, Via Mario Negri 2, 20156 Milan, Italy

**Keywords:** Classification of STAD tumors, Cell lines, Claudin^low^, NF-YA

## Abstract

**Background:**

Claudin-3, Claudin-4, and Claudin-7 are expressed on the surface of epithelial cells. Their absence in neoplastic cells of epithelial origin is an aggressiveness marker in different cancers. The *NF-YA* gene codes for the *Nuclear-Transcription-Factor-Y-Subunit-A*, which is overexpressed in various tumors. In tumors, the relative ratio of the two major *NF-YA* alternative splicing isoforms, *NF-YA long* and *NF-YA short*, is associated with a mesenchymal phenotype and a poor prognosis. Based on a high *NF-YA long*/*NF-YA short* ratio, we generated a 158-gene signature that is common to Claudin^low^ Breast Carcinomas (BRCA) and Stomach Adenocarcinomas (STAD).

**Methods:**

To better classify STAD Claudin^low^ tumors, we employed a hierarchical clustering approach based on our 158-gene signature to classify STAD into a Claudin^low^ subgroup. We tested the classification potential of our signature in TCGA as well as in two additional datasets of tumors. We used the deep-learning *DeepCC* tool and the 158-gene signature to classify the STAD cell lines available in the CCLE platform. Obtained data were validated with qRT-PCR and Western blots.

**Results:**

The 158-gene signature resulted in the selection of a STAD subgroup with effective Claudin^low^ expression and with a high *NF-YA long*/*NF-YA short* ratio. This Claudin^low^ subgroup was separated from the EMT subgroup of STAD and it is characterized by poor clinical outcome. We identified nine Claudin^low^ STAD cell lines with a high *NF-YA long*/*NF-Y short* ratio and validated the expression of selected markers.

**Conclusions:**

Our work supports the notion that three overlapping features—low expression of Claudin-3/4/7, high *NF-YA long*/*NF-YA short* ratio and a 158-gene signature—mark a specific subset of STAD characterized by mesenchymal features and poor prognosis.

**Supplementary Information:**

The online version contains supplementary material available at 10.1007/s10120-025-01671-1.

## Introduction

Stomach Adenocarcinoma (STAD) is a heterogeneous type of tumor characterized by a poor survival rate [1]. The Lauren’s classification groups gastric cancers into intestinal (*IT*), diffuse (*DF*), and mixed (*MX*) subtypes according to their histological features [2, 3]. In addition, gastric cancers can be classified on the basis of specific genetic mutations, chromosomal alterations, epigenomic features, and gene expression patterns [4–7]. The TCGA-based (The Cancer Genome Atlas) molecular classification of gastric cancers [8] defines four distinct subtypes (Reviewed in [9–11]), *i.e.*, EBV (*EBV infected*), MSI (*MicroSatellite Instability*), GS (*Genomically Stable*), and CIN (*Chromosomal Instability*) tumors. Similarly, the ACRG-based (*Asian Cancer Research Group*) molecular classification identifies an equal number of stomach tumor subtypes, i.e., MSS;TP53^−^ (*MicroSatellite Stable; TP53 inactive*), MSS/TP53^+^, MSI, and EMT (*Epithelial to Mesenchymal Transition*) [12].

Claudins are a large family of proteins which are expressed on epithelial cells and are involved in ordered cell–cell interactions [13]. The EMT process reduces the levels or abolishes the expression of certain Claudins via transcriptional silencing of the corresponding genes [14]. Low expression of Claudin-3, Claudin-4, and Claudin-7 (*CLDN3*, *CLDN4*, and *CLDN7*) is associated with 3 types of epithelial cancers: breast cancer (BRCA), STAD, and bladder cancer (BLCA). In BRCA, tumors with low expression of Claudins were originally included in the large Basal-like or Triple-Negative, TNBC subgroup. Additional work led to the identification of a further subgroup of tumors characterized by distinct molecular features and a transcriptomic signature consisting of 437 genes [15]. Subsequent studies reduced the members of this signature to 19 genes [16]. Claudin-4 is frequently overexpressed in STAD, and membranous Claudin-4 expression correlates with better prognosis and reduced cancer cell migration and invasion, while also enhancing tight junction barrier function [17]. A fifth STAD subgroup (Claudin^low^) was added to the four types of tumors mentioned above [8, 12]: this additional subgroup was identified on the basis of a 24-gene transcriptomic signature, which was obtained by analysis of the EMT tumors available in the ACRG classification [18]. In the case of BLCA, a separate Claudin^low^ subgroup was identified and reported in the scientific literature. This BLCA subgroup is associated with a 40-gene transcriptomic signature known as BCL40 [19, 20].

In the BRCA context, the presence of a distinct Claudin^low^ subgroup was challenged on the basis of the following reasons: (i) aggressive Claudin^low^ BRCAs arise not only from mesenchymal Basal-like tumors, but also from Luminal tumors, which are generally characterized by epithelial features [21]; (ii) unlike the Basal-like/TNBC counterparts, most Claudin^low^ tumors show Estrogen-Receptor (*ER*) expression, which is a hallmark of Luminal cancers [22]; (iii) immunohistochemistry (IHC) studies on *CLDN3*, *CLDN4*, and *CLDN7* lack univocal correlations between low expression of the 3 Claudins and clinical features or EMT-markers levels (Reviewed in [13]); (iv) often, co-localization of Claudins on the cellular membrane and inside the cell is inconsistent. Overall, the results obtained in BRCA challenge the presence of a distinct Claudin^low^ subgroup in STAD as well.

*NF-Y* is a trimeric transcription factor (*TF*) playing an important role in the regulation of various genetic *loci*, with particular reference to those involved in the cell cycle and metabolism [23]. We characterized the expression of the *NF-Y* subunits in epithelial cancers using a systematic approach [23]. In particular, we reported data on the differential regulation of two alternatively spliced isoforms of *NF-YA*, *NF-YAl* (*NF-YA long*) and *NF-YAs* (*NF-YA short*), in aggressive cancers. Our data demonstrate a high *NF-YAl*/*NF-YAs* expression ratio in the EMT subgroups of BRCA, STAD, and Head and Neck Squamous Cell Carcinoma (HNSCC). With the use of the *NF-YAl*/*NF-YAs* expression ratio, we analyzed the STAD and BRCA cases available in the TCGA database, and we identified a common 158-gene signature predicting poor clinical outcomes [24]. We also deconvoluted the single-cell RNA-sequencing (scRNA-seq) data, which allowed us to predict the EMT features of the neoplastic cells within each tumor. In a series of experimental studies, we ablated *NF-YA* exon-3 in clones derived from Claudin^low^ BRCA cell lines with a genome editing approach. This resulted in cellular clones characterized by the sole expression of the “epithelial” *NF-YAs* splicing variant. The genetically modified cells showed normal growth, although they presented an impairment of the mesenchymal/migratory features *in vitro* and metastatic behavior *in vivo* [24]. Thus, at least in BRCA cells, the relative levels of the *NF-YA* isoforms play a relevant role in shaping the EMT phenotype.

In the present study, we compare our 158-gene signature and the 24-gene signature described by Nishijima et al. [18] to identify a Claudin^low^ subtype in STAD tumors. In addition, we provide an improved classification of the STAD cell lines available in the CCLE (*Cancer Cell Line Encyclopedia*) database.

## Materials and methods

### STAD RNA-seq data and molecular classifications

We downloaded the STAD gene expression data available in the TCGA dataset from the http://firebrowse.org/ web page, which is contained in the *illuminahiseq_rnaseqv2-RSEM_genes* and *illuminahiseq_rnaseqv2-RSEM_isoforms* files. As of March 2025, RNA-seq data for 415 primary tumor samples were available. We retrieved the FASTQ files associated with: (i) 50 STAD cell lines available in the CCLE (*Cancer Cell Line Encyclopedia*, SRA BioProject PRJNA523380) database and an independent dataset (SRA BioProject PRJNA327709). When duplicate samples were available in the 2 datasets, we performed our analyses on the CCLE data; (ii) 231 STAD primary tumor samples and 230 paired normal tissues from Harbin Medical University Cancer Hospital (SRA BioProject PRJNA764173) [25]; (iii) 60 STAD primary tumor samples from Seoul National University Hospital (SRA BioProject PRJNA1119255); (iv) 13 STAD primary tumor samples available inside the Istituto di Ricerche Farmacologiche Mario Negri IRCCS (EMBL-EBI Annotare accession E-MTAB-12385 and SRA BioProject PRJEB57792) [26]; (v) 12 STAD primary non-metastatic tumor samples and 5 STAD tumors with peritoneal metastasis from The Fourth Hospital of Hebei Medical University (SRA BioProject PRJNA1220682) [27]. These data were used to define mRNA expression with the use of RSEM-1.3.3 [28]. For the STAD primary tumor samples of the TCGA, we initially applied the classification outlined in [29]. For the CCLE cell lines, we used the classification available in [30] as a starting point for our re-classification procedure. The WHO International Classification of Diseases for Oncology, 3rd Edition (ICD-O-3, https://www.who.int/standards/classifications/other-classifications/international-classification-of-diseases-for-oncology) was downloaded from the cBioPortal (https://www.cbioportal.org/).

### Hierarchical clustering and Claudin^low^ subtype re-classification of the TCGA STAD tumors

We applied a variance-stabilizing transformation (*vst*) on gene raw count data of the STAD tumors using the *DESeq2 R* package [31]. Subsequently, we performed hierarchical clustering with our 158-gene signature [24] using *SigClust2* (v1.2.4) [32], applying the *Ward.D2* linkage and the *manhattan* metric. The alpha parameter was set to 0.05, and significance at each parent node was assessed using the built-in *FWER* control procedure before testing daughter nodes.

### Differential gene expression and functional enrichment analyses

Differential gene expression analysis of TCGA subtypes was performed using *DESeq2* R package. We used expression fold change (log2FC) to denote upregulation or downregulation, while FDR < 0.01 and |log2FC|> 1 were set as inclusion criteria for DEG selection.

We used DAVID [33] (GO_Direct categories) and the Enrichr website (https://maayanlab.cloud/Enrichr) for Gene Ontology (GO) terms and Reactome Pathways enrichment analyses, respectively. The *UpSetR* package [34] was used to generate the upset plots shown in Fig. 2, while the heatmap was produced with pheatmap (https://github.com/raivokolde/pheatmap). The volcano plot in Supplementary Fig. 2 was created with the EnhancedVolcano package (10.18129/B9.bioc.EnhancedVolcano).

### Prediction of STAD tumors stromal and immune cells infiltration

We assessed the Immune and Stromal scores of STAD primary tumors using the *tidyestimate* package (version 1.1.1, https://github.com/KaiAragaki/tidyestimate), starting from gene-level expression data in the form of log2(TPM + 1).

### Analysis of STAD survival data

We retrieved Overall and Progression-Free Survival (OS and PFS, respectively) time records of STAD patients from the http://xena.ucsc.edu/ web page [35]. We stratified all the tumors for which PFS records were available according to the novel classification proposed here. Survival analysis was performed according to the Kaplan–Meier analysis and log-rank test.

### Re-classification of the STAD cell-lines

We first extended the original classification of the cell lines [30] to all the available studies using *DeepCC* [36], run with the log_2_(TPM + 1) value as input. Next, we conducted ssGSEA (single sample Gene Set Enrichment Analysis) [37] employing our 158-gene signature. We classified cell lines showing a *z-score* normalized enrichment > 1 as Claudin^low^.

### Cell cultures

Human gastric cell lines were cultured under standard *ATCC* or *Riken Cell Bank* conditions. HS746T, LMSU, MKN7, NCIN87, MKN45, and AGS cells were grown in RPMI (Euroclone ECB9006L) supplemented with FBS (Euroclone ECS0165L) at 10%. All cell line media were supplemented with 1 mM L-glutamine (Euroclone ECB3000D) and 100 μg/mL penicillin/100 μg/mL streptomycin (Euroclone ECB3001D).

### RNA extraction and retro-transcription

RNA was isolated using the Tryzol (Sigma Aldrich T9424) protocol. Pellets (3 × 10^6^ cells) were resuspended and lysed in 1 mL of Tryzol. After lysis, 200 µL of chloroform was added and samples were vortexed for 15″. Following a 5’ incubation at room temperature, samples were centrifuged at 12,000 × g at 4 °C for 15’, isopropanol (500 μL) was added to the RNA-containing aqueous phase and the tubes were inverted gently. After centrifugation as above, the supernatant was removed and the RNA pellet was washed by the addition of 70% RNAse-free ethanol (500 μL). RNA pellets were resuspended in DEPC-treated water. We quantified the concentration of RNA with Nanodrop and we determined RNA integrity by agarose gel electrophoresis. As for retro-transcription, total RNA (1 μg) was reverse transcribed using M-MLV SuperMIX 5X + M-MLV mix (Genespin STS-MSX) according to the Manufacturer’s instructions. The cDNAs were diluted 1:5 in TE (Tris 10 mM pH 7.5, EDTA 1 mM).

### qRT-PCR and western blot analyses

Real-time PCR was performed using the BIO-RAD CFX ConnectTM RT-PCR system with oligonucleotides designed to amplify 100–200 bp fragments. The housekeeping gene Rps20 was used to normalize the expression data. The relative sample enrichment was calculated with the formula 2–(ΔΔCt), where ΔΔCt = [(Ct sample – Ct Rps20)x − (Ct sample – Ct Rps20)y], x = sample and y = sample control. The set of primers used is described in Supplementary Table 1.

For the preparation of whole-cell extracts, cells were resuspended in RIPA buffer (10 mM TrisHCl pH 8.0, 1 mM EDTA, 0.5 mM EGTA, 0.1% SDS, 0.1% sodium deoxycholate, 140 mM NaCl, 1% Triton X-100, 1 mM PMSF, Protease inhibitor cocktail) and incubated for 30’ on ice. Samples were centrifuged at 13,000 rpm for 10 min at 4 °C, and the supernatant was recovered and quantified using the Bradford protein assay. 30 µg of extracts were loaded on a 12% SDS–polyacrylamide gel and analyzed by Western blot with anti-Vinculin (Sigma-Aldrich 05–386), anti-β-tubulin (Sigma-Aldrich T5293), anti-*NF-YA* G2 (Santa Cruz Biotech., sc-17753), anti-CDH1 (BD Biosciences., 610,181), and anti-Vimentin (Cell Signaling., D21H3) primary antibodies. Secondary antibodies were peroxidase-conjugated anti-mouse (A4416 Sigma-Aldrich) and anti-rabbit (A6154 Sigma-Aldrich). Detection was performed with ChemiDoc (Bio-Rad) and exported using the Image-Lab software (Bio-Rad).

## Results

### A novel classification of Claudin^low^ STADs with the use of the 158-gene signature based on the *NF-YAl*/*NF-YAs* expression ratio

We previously classified all STAD tumors in the TCGA database according to the 4 TCGA and ACRG subtypes [29] and complemented this with a fifth Claudin^low^ subtype defined on the basis of the 24-gene transcriptomic signature described by Nishijima [18].

The number of Claudin^low^ tumors (Claudin^low^: 79) and the other 4 STAD tumor subtypes (EMT:61; MSI:82; MSS/TP53^−^:104; MSS/TP53^+^:73) are comparable [24]. As expected, the predicted Claudin^low^ tumors express significantly lower amounts of *CLDN7* relative to the MSI, MSS/TP53^−^, and MSS/TP53^+^ (Fig. S1). A similar situation is observed when *CLDN4* levels in Claudin^low^, MSI, and MSS/TP53^−^ tumors are considered. Surprisingly, no statistically significant difference in the levels of *CLDN3*, *CLDN4*, and *CLDN7* mRNAs is detected in Claudin^low^ and EMT tumors. In addition, Claudin^low^, EMT, MSI, MSS/TP53^−^, and MSS/TP53^+^ tumors show no difference in *CLDN3*. Overall, our data indicate that the Claudin^low^ denomination applied to the subgroup of STAD tumors defined by the 24-gene signature is incorrect, as it does not reflect the *CLDN3*, *CLDN4*, and *CLDN7* expression levels appropriately.

**Fig. 1:**
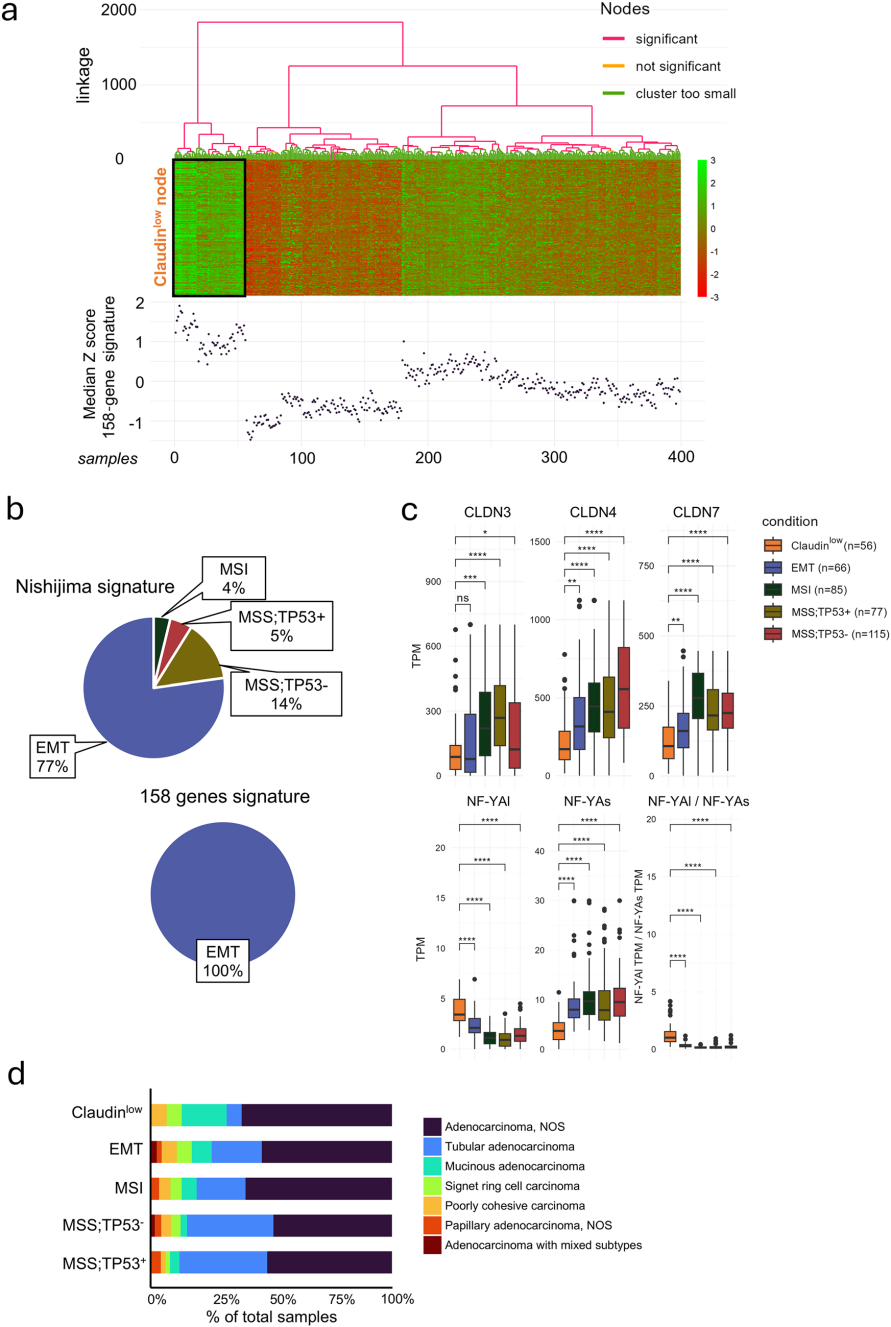
Re-definition of the Claudin^low^ subtype of the TCGA STAD tumors. **a** *SigClust2* hierarchical clustering of all STAD tumors of TCGA according to our 158-gene signature. Top: heatmap depicting the expression of signature genes within the tumor groups defined by hierarchical clustering. The proposed Claudin^low^ node is highlighted by a black square. Bottom: the dotplot shows an aggregate view of the signature-genes expression in STAD tumors, as calculated by the median *z-score*. **b** Proportion of samples included in the previous (*n* = 79, Left) and current (*n* = 56, Right) Claudin^low^ groups according to their ACRG classification. **c** Box plots showing the *CLDN3*, *CLDN4*, *CLDN7*, *NF-YAl* and *NF-YAs* expression levels as well as the *NF-YAl*/*NF-YAs* expression-ratio values of the STAD tumors in the TCGA dataset. The values are shown in CCLE (*Transcripts Per Million*). Tumor types are defined according to the new molecular classification. **d** Barplot showing ACRG + Claudin^low^ subtypes distribution into WHO ICD-O-3 categories for gastric cancer, expressed as percentages of total samples within each subtype. NOS = not otherwise specified

Interestingly, our transcriptomic analyses indicate that the so-called Claudin^low^ subgroup can be separated from all the other STAD tumor subtypes with the use of the *NF-YAl*/*NF-YAs* expression ratio. Indeed, the *NF-YAl*/*NF-YAs* median values are significantly higher in Claudin^low^ tumors relative to the EMT, MSI, MSS/TP53^−^, and MSS/TP53^+^ counterparts (Fig. S1). On the basis of this observation, we used the 158-gene signature [24], derived by WGCNA (Weighted Gene Co-expression Network Analysis), as associated with a high threshold value of the *NF-YAl*/*NF-YAs* ratio in both BRCA and STAD tumors (Supplementary Table 2). A hierarchical clustering of STAD samples based on the 158-gene signature permits a novel definition of the Claudin^low^ subset, reducing the original number of Claudin^low^ tumors from 79 to 56 (Supplementary Table 3 and Fig. 1a). According to the ACRG classification, most of the 79 Claudin^low^ tumors based on the Nishijima classification originate from the EMT subgroup, while a minor portion derives from other subgroups (Fig. 1b). By contrast, the new classification indicates that all newly identified 56 Claudin^low^ tumors originate from the EMT subgroup of STADs (Fig. 1b). Figure 1c shows the expression boxplots of the mRNAs coding for *CLDN3*, *CLDN4*, *CLDN7*, *NF-YAl*, *NF-YAs*, and *NF-YAl*/*NF-YAs* values in our panel of STADs classified according to the 158-gene signature. As expected, Claudin^low^ tumors show significantly lower levels of the *CLDN3*, *CLDN4*, and *CLDN7* mRNAs relative to the other subgroups. The exception is represented by *CLDN3*, whose average expression levels are the same in Claudin^low^ and EMT tumors. In addition, the Claudin^low^ specimens present the expected increase of the *NF-YAl*/*NF-YAs* ratio, due to a statistically significant increase in *NF-YAl* levels and a corresponding decrease in *NF-YAs* (Fig. 1c). Looking at WHO ICD-O-3 categories (International Classification of Diseases for Oncology, 3rd Edition, https://www.who.int/standards/classifications/other-classifications/international-classification-of-diseases-for-oncology), we noticed that Claudin^low^ tumors were significantly enriched in the mucinous adenocarcinoma fractions (n = 6 out of 18 total, Fisher exact test *p* value = 1.12 × 10–2), while including a smaller number of tubular adenocarcinomas (*n* = 2 out of 71, *p* value = 2.29 × 10–3) (Fig. 1d). Mucinous adenocarcinomas are characterized by an increased invasion capacity, and they are localized in the upper part of the stomach [38].

In conclusion, our results support the appropriateness of the 158-gene signature to identify the Claudin^low^ subset of gastric cancers.

### Patterns of genes differentially expressed in the Claudin^low^ subset of gastric tumors

**Fig. 2:**
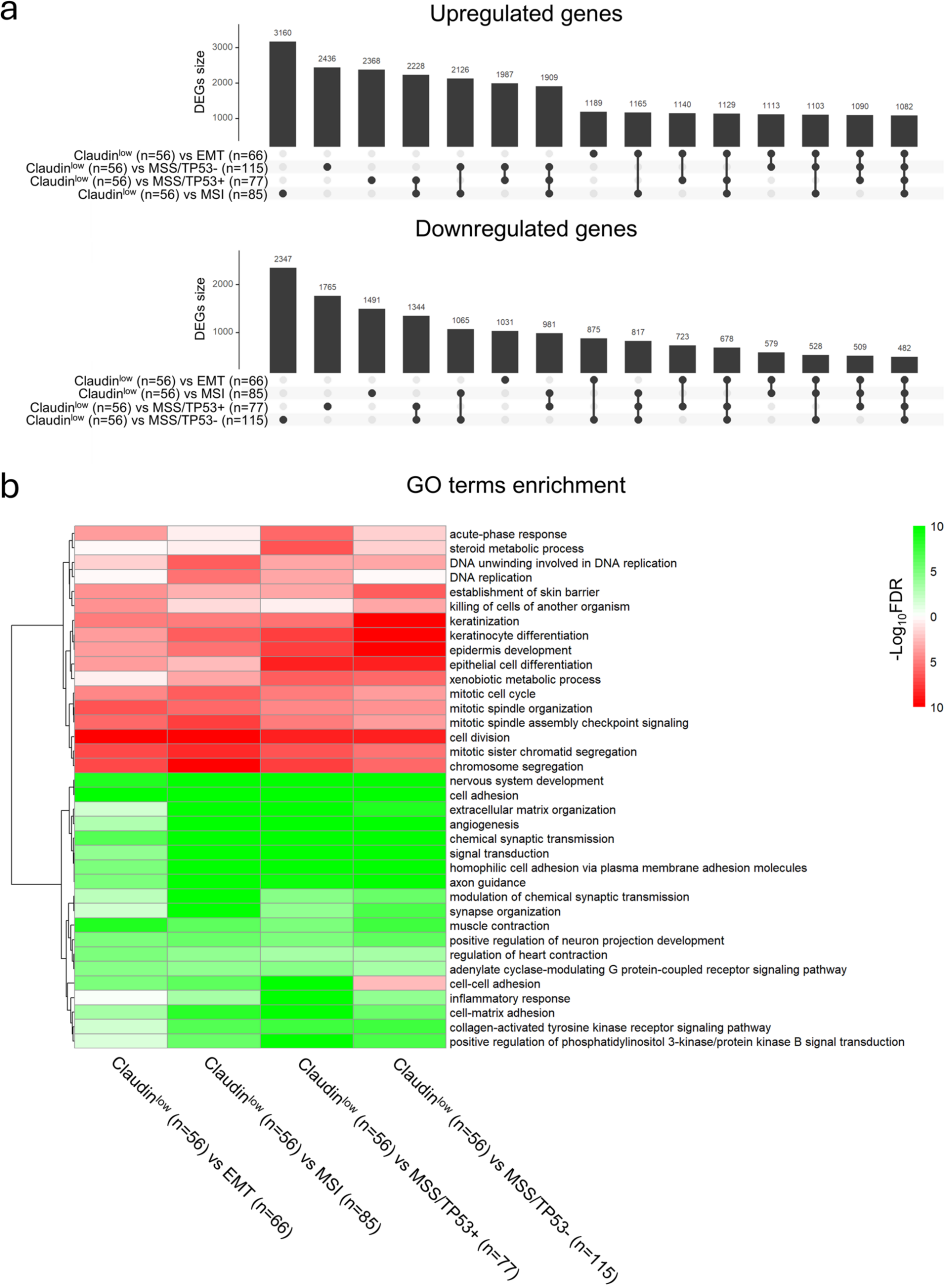
Differentially Expressed Genes in Claudin^low^ STAD tumors showing a high *NF-YAl*/*NF-YAs* expression ratio. **a** The upper plots show the number of upregulated (Top) and downregulated (Bottom) genes in Claudin^low^ samples as compared to the other STAD subtypes. In addition, the Panel illustrates each possible intersection among the different comparisons. **b** The heatmap depicts the enrichment of the Gene Ontology (GO) terms in upregulated (green) and downregulated (red) genes determined by the comparisons shown in (**a**)

We used RNA-seq data from the TCGA database to identify DEGs (Differentially Expressed Genes) in the Claudin^low^ subset of STADs with a “pairwise-comparisons” approach (Supplementary Table 4). The numbers of upregulated and downregulated DEGs determined in each comparison of subsets are shown in Fig. 2a. The minimum number of exclusive DEGs is observed in the Claudin^low^/EMT match (1189 upregulated and 1031 downregulated genes). The other Claudin^low^ matches show a higher number of exclusive DEGs (> 2000 upregulated genes and > 1491 downregulated genes). The upregulated and downregulated DEGs emerging from each comparison can be grouped under several GO (Gene Ontology) terms whose enrichment is characterized by a different degree of significance (Fig. 2b). As for the down-regulated DEGs, the most significant enrichment values are observed for terms describing various mitotic processes. It is worthwhile mentioning that all the comparisons show a significant enrichment of downregulated genes involved in developmental processes, such as *establishment of skin barrier*, *keratinization*, *epidermis development* and *epithelial cell differentiation*. This is particularly relevant for the comparisons with the MSS/TP53^−^ and MSS/TP53^+^ subtypes, which are characterized by an epithelial phenotype. As for the upregulated DEGs, the enrichment in genes participating in processes such as *muscle contraction*, *angiogenesis* and *cell–cell adhesion*, which are typical of cells with a mesenchymal phenotype, is of major interest: these processes are particularly enriched in the comparison between the Claudin^low^ and the EMT tumor subtypes.

To better define differences of the 5 subgroups in TCGA, and verify partitioning of the EMT and Claudin^low^ clusters, we performed Principal Component Analysis (PCA), and the results are shown in Fig. S2a: while the epithelial subgroups are largely clustered together, EMT samples are distinct, and Claudin^low^ samples are further separated. Fig. S2b shows a Volcano plot of the differences in DEG between EMT and Claudin^low^ samples, with several genes showing a clear differential expression between the two subgroups. This is reflected in the Gene Ontology and Reactome Pathways plots of up- and down-regulated genes in the two cohorts (Fig. S2c), showing highly enriched terms of *cell adhesion* and *muscle contraction* in Claudin^low^, and *cell division* and *mitotic cell cycle* in EMT.

### Use of the 158-gene signature to classify additional STAD cases

**Fig. 3:**
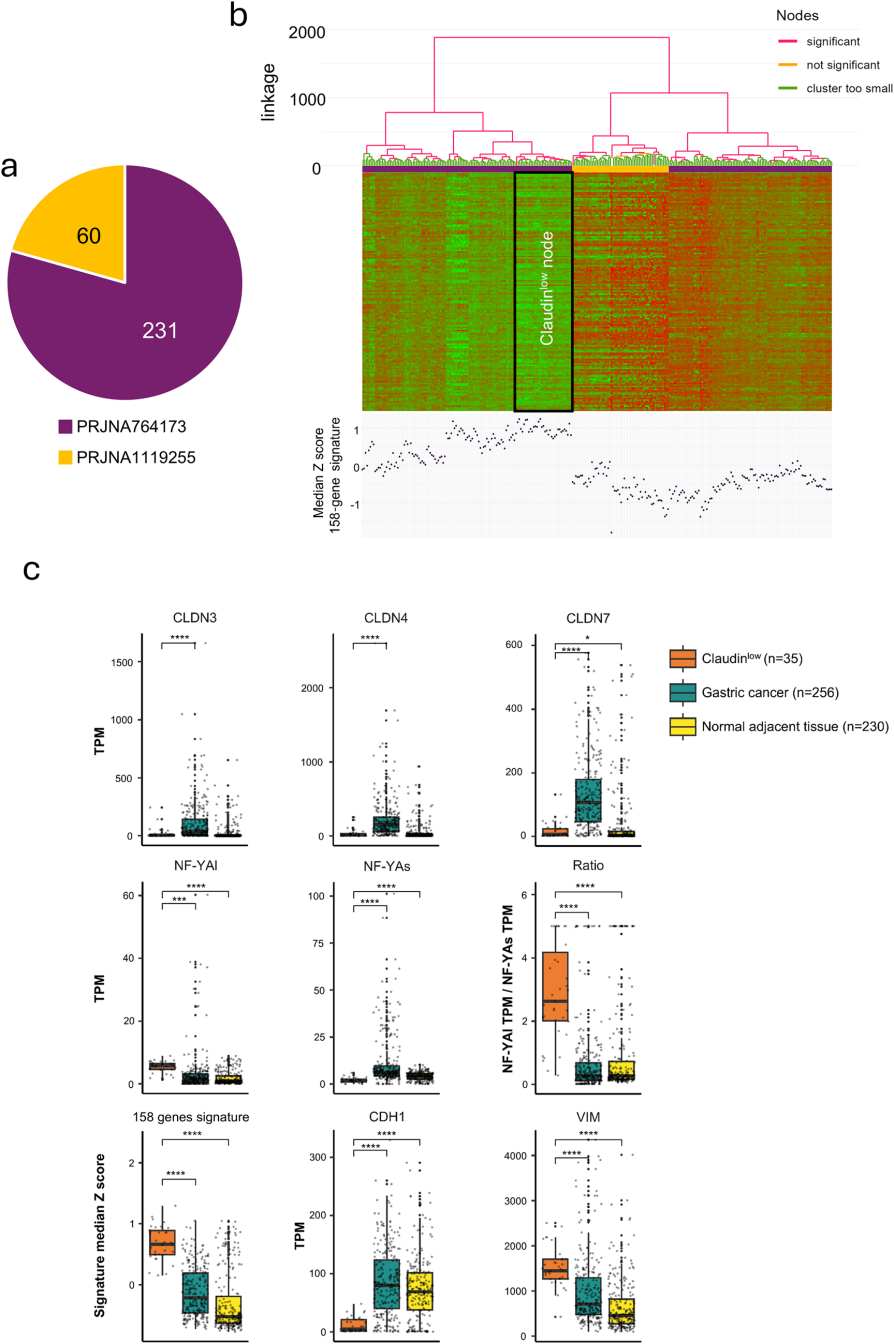
Claudin^low^ classification in additional primary tumor datasets. **a** Pie chart of the gastric cancer samples collected from two independent sources, corresponding to the SRA accessions PRJNA764173 and PRJNA1119255. **b** *SigClust2* hierarchical clustering of primary STAD samples detailed in **a** according to our 158-gene signature. Top: heatmap showing the expression of signature genes across tumor groups identified by hierarchical clustering, with the proposed Claudin^low^ node outlined in black. Bottom: Dot plot summarizing the expression of signature genes in STAD tumors, represented by the median *z-score*. **c** Box plots display the expression levels of *CLDN3*, *CLDN4*, *CLDN7*, *NF-YAl*, and *NF-YAs*, along with the *NF-YAl/NF-YAs* expression ratio, the correlation with the 158-gene signature, and the marker genes *CDH1* and *VIM* in STAD tumors from the independent datasets PRJNA764173 and PRJNA1119255. Expression values are reported in TPM (Transcripts Per Million), while correlations with the 158-gene signature are represented as median *z-scores*

We wished to verify the TCGA results using two additional RNA-seq datasets, PRJNA764173 of 231 and PRJNA1119255 of 60 patients, both of Asian origin (Fig. 3a). Exploiting the 158-gene signature, hierarchical clustering of the 291 samples yielded a Claudin^low^ node with 35 samples (Fig. 3b). We remark that the incidence of this cluster −12% in these datasets is similar to the Claudin^low^ cluster in TCGA – 13%. These samples show low expression of Claudins and of the epithelial marker E-Cadherin, high levels of *NF-YAl* and *NF-YAl/NF-YAs* ratio, as well as of the mesenchymal marker Vimentin (Fig. 3c). Finally, we used our 158-gene signature to characterize 13 Italian patients (PRJEB43867) [25]: median *z-score* values are positive in 7 cases, negative in 4 cases, and are close to 0 in 2 cases (GC15 and GC6) (Fig. S3a). The 7 cases characterized by a positive *z-score* value and GC15 present with high levels of *NF-YAl* and low levels of *NF-YAs*, which results in a high *NF-YAl/NF-YAs* value (Fig. S3a). With the exception of *CLDN3* in GC15, the expression of *CLDN3*, *CLDN4*, and *CLDN7* is low in all these samples (Fig. S3b). Altogether, these results confirm the presence of a subgroup of STAD that has low expression of Claudin-3/-4/-7.

### Claudin^low^ samples features unique tumor-stromal interactions

**Fig. 4:**
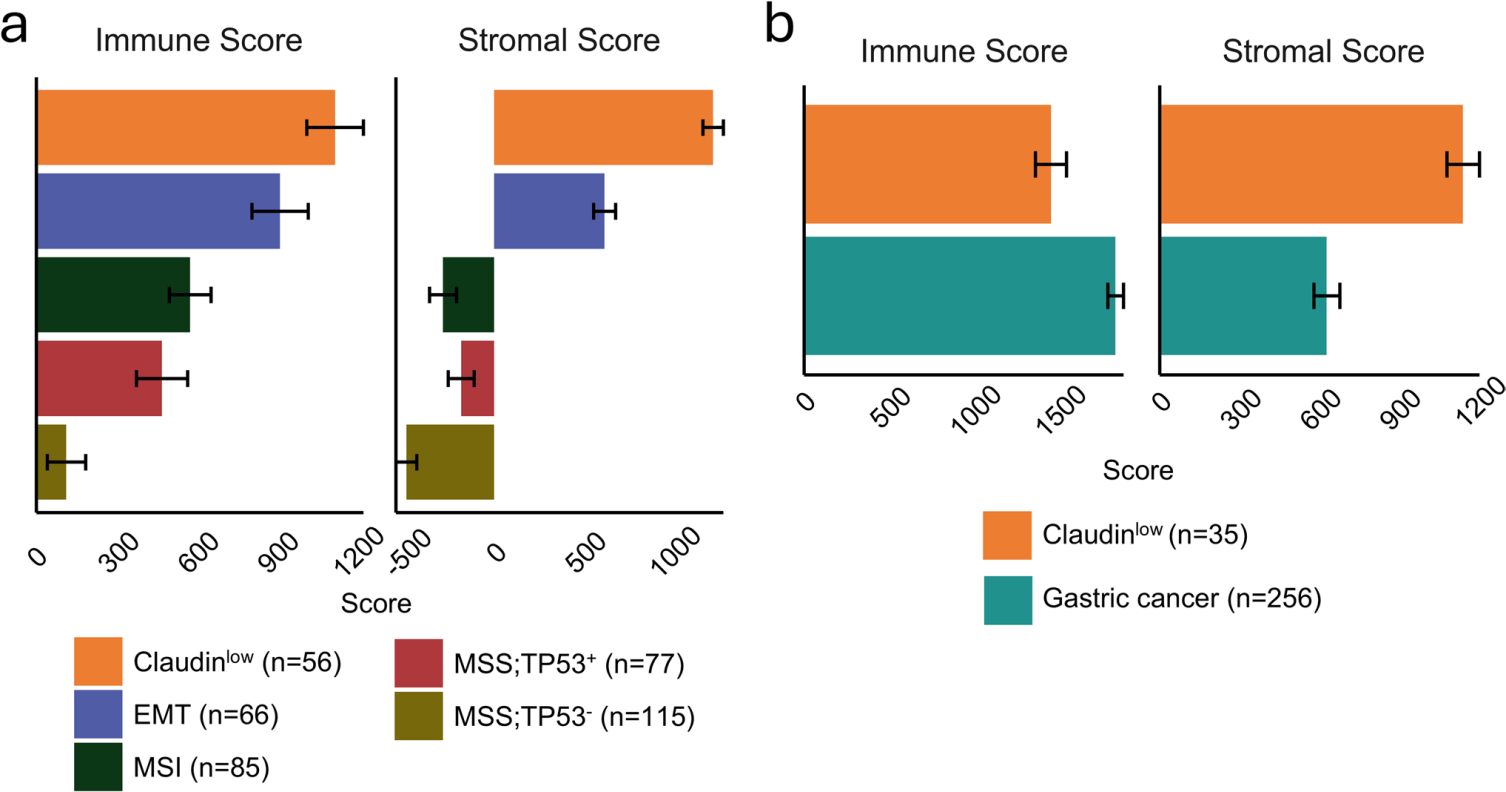
Claudin^low^ gastric tumors show distinct microenvironments. **a** Immune score and stromal score estimated for each gastric cancer subtype. Claudin^low^ and EMT tumors display the highest immune and stromal infiltration scores. **b** Comparison of Claudin^low^ tumors with the broader gastric cancer cohort from the PRJNA764173 and PRJNA1119255 datasets, highlighting elevated stromal scores in Claudin^low^ tumors relative to the general gastric cancer population. Error bars represent standard errors of the mean

In BRCA, Claudin^low^ tumors were initially described as exhibiting a pronounced immune and stromal cell infiltration [39]. We decided to assess tumor microenvironment composition with the ESTIMATE algorithm, which employs single-sample Gene Set Enrichment Analysis (ssGSEA) to assign an Immune Score and Stromal Score to each tumor sample based on RNA-seq gene expression [40]. Applying this strategy, we discovered that TCGA STAD Claudin^low^ tumors were characterized by increased Immune and Stromal Scores compared to epithelial subtypes, while EMT showed intermediate values in the two metrics (Fig. 4a).

In the second and third dataset of primary gastric cancers, Claudin^low^ classified samples had a higher Stromal Score than other samples, but a lower Immune Score (Fig. 4b). Together, these results suggest that Claudin^low^ gastric tumors share with their breast cancer counterparts a distinctive microenvironmental profile marked by elevated stromal content, while immune infiltration appears more variable across datasets.

### Insights into the prognostic value of the newly identified subgroup of Claudin^low^* STAD*s with a high *NF-YAl*/*NF-YAs* expression ratio

**Fig. 5:**
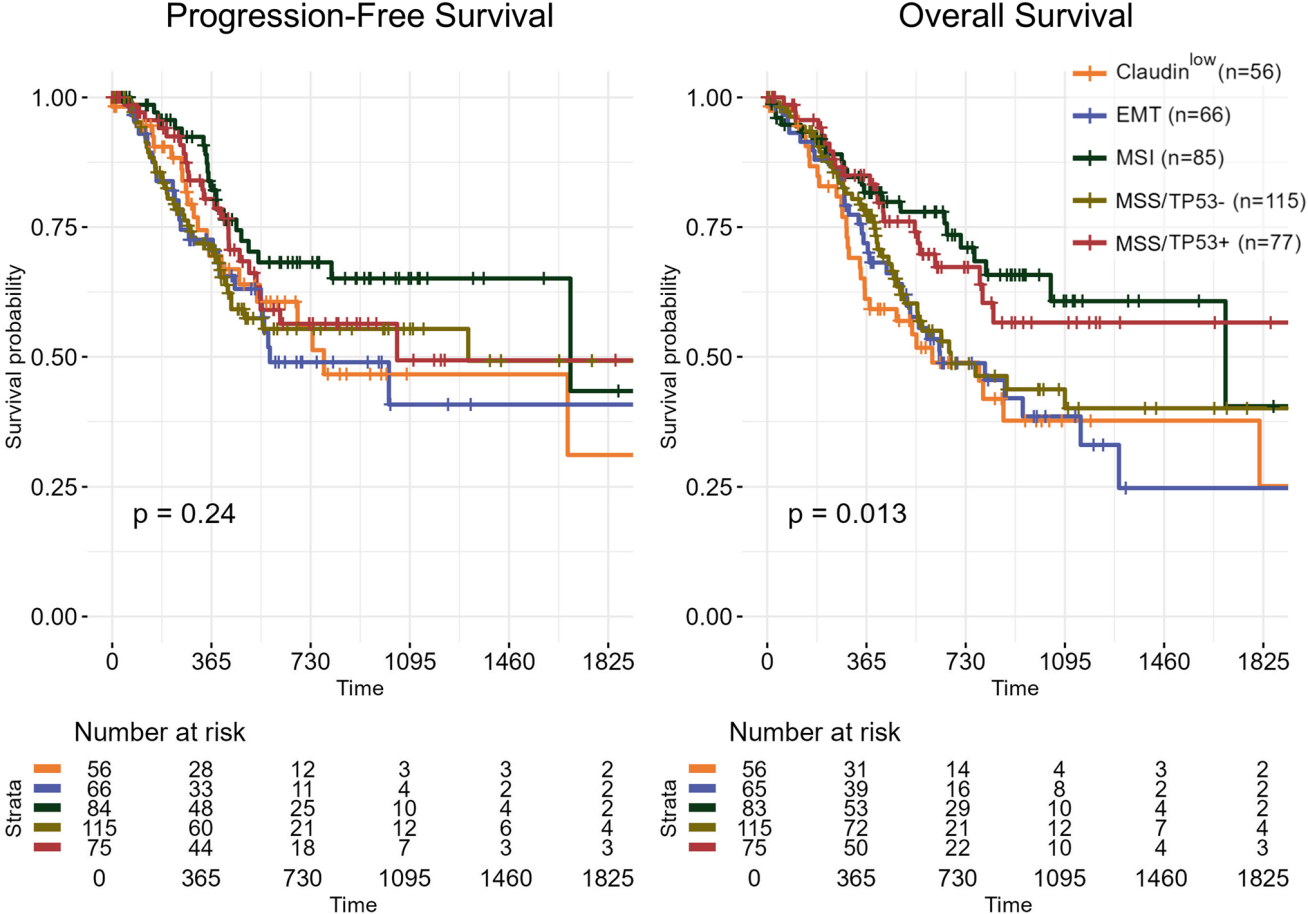
Clinical outcome of the Claudin^low^ STAD tumors showing a high *NF-YAl*/*NF-YAs* expression-ratio. The Figure shows the Kaplan–Meier survival curves of STAD patients. The Progression Free Survival values (Left) and Overall Survival values (Right) across the STAD molecular subtypes are illustrated. The *p*-values were determined using the log-rank test

**Fig. 6:**
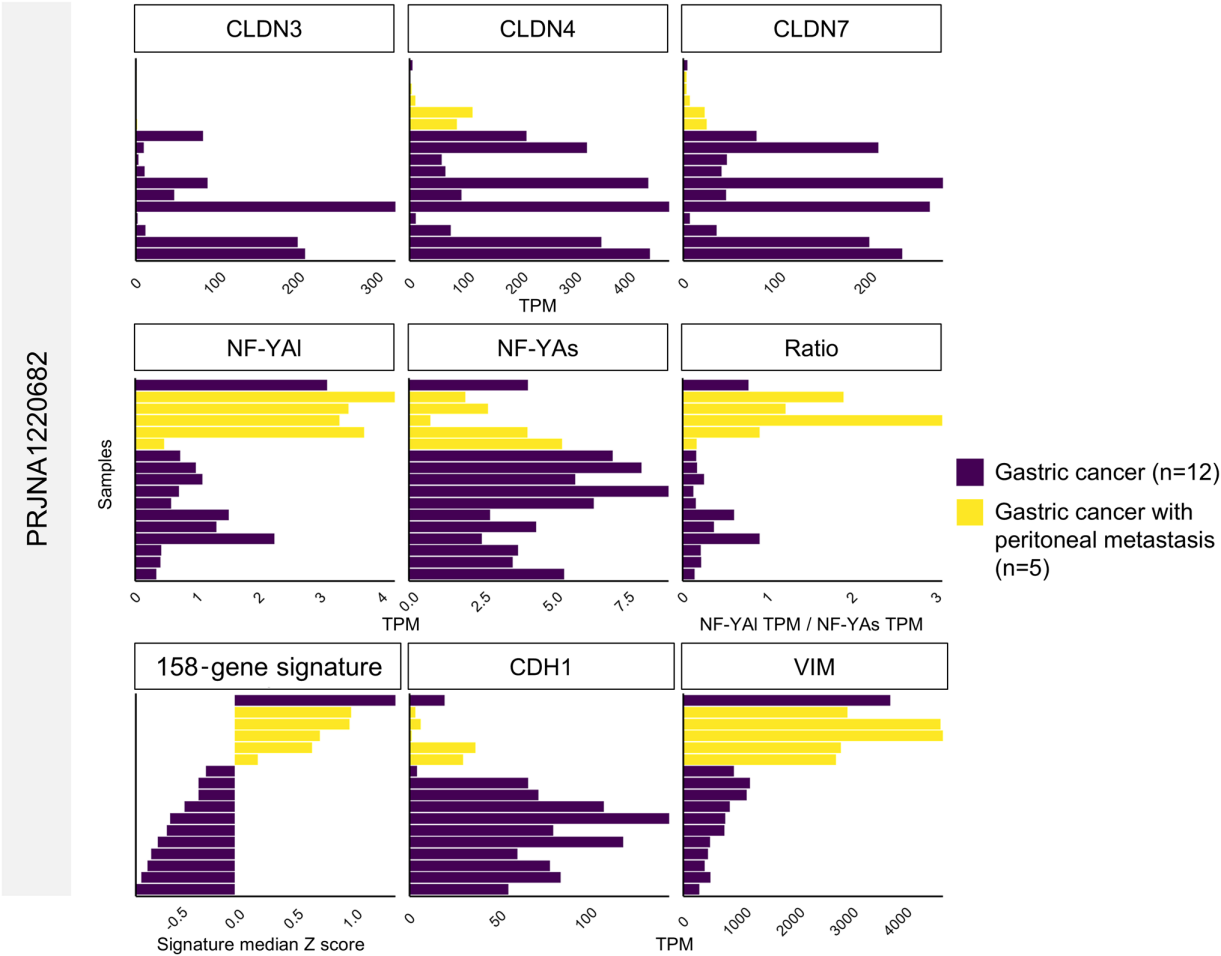
The 158-gene signature strongly correlates with gastric cancer that originated peritoneal metastasis. Bar plots show expression of *CLDN3*, *CLDN4*, *CLDN7*, *NF-YAl*, and *NF-YAs*, the *NF-YAl/NF-YAs* expression ratio, the 158-gene signature score, and the epithelial and mesenchymal markers *CDH1* and *VIM* in individual samples of gastric cancer with or without peritoneal metastasis. Expression data are presented as Transcripts Per Million (TPM), while 158-gene signature values are expressed as median *z-scores*

To obtain insights into the progression/mortality rates of the 56 Claudin^low^ tumors and the other subgroups, we performed an evaluation of the clinical data available in the TCGA database. In particular, we compared the PFS (Progression-Free Survival) and the OS (*Overall-Survival*) curves of the Claudin^low^ subgroup of patients with the EMT, MSI, MSS/TP53^−^, and MSS/TP53^+^. The Kaplan–Meier PFS curves demonstrate that there are no statistically significant differences, whereas the OS curves indicate that Claudin^low^ shows a significantly lower survival rate/probability than MSS/TP53^+^ and MSI patients (Fig. 5). On the other hand, there is a lack of significant differences between Claudin^low^ and EMT cases, both marked by “mesenchymal” phenotypes. Thus, we conclude that the prognoses of Claudin^low^ and EMT tumors are the worst. Unfortunately, there are no clinical data available for the 231 and 60 patients of PRJNA764173 and PRJNA1119255 classified above. To investigate further the markers above, specifically in Grade IV tumors, we interrogated an available RNA-seq dataset (PRJNA1220682) of gastric cancer with or without peritoneal metastasis. The paucity of the samples precludes the definition of clustering as in Fig. 1 and Fig. 3, but the gene expression data of Fig. 6 shows that, bar one exception, gastric cancers that produced a distant metastasis—the hallmark of stage IV cancer—have lower Claudins and E-Cadherin levels, higher *NF-YAl*, *NF-YAl/NF-Ys *ratios, and Vimentin.

### A novel classification of the STAD cell lines

The bulk RNA-seq generated from the TCGA, 291 stomach-tumors dataset and our cohort of Italian patients define genes expressed not only in the neoplastic cells, but also in tumor-associated immune, stromal, and endothelial cells. Thus, it is important to perform the same type of analyses on STAD cell lines of the CCLE portal for which RNA-seq data are available.

**Fig. 7:**
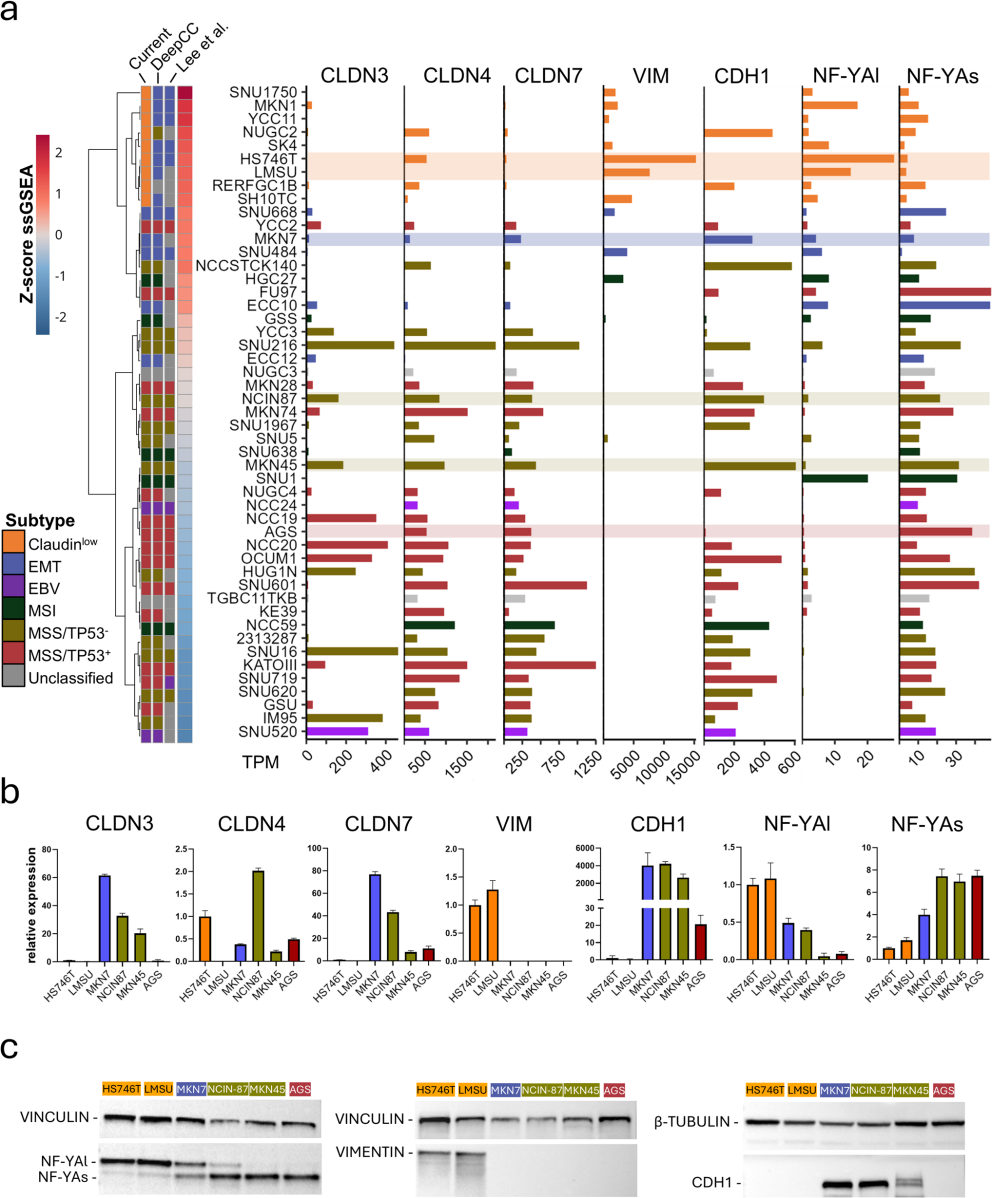
A novel classification and characterization of STAD cell lines based on our 158-gene signature and the expression of the *CLDN3*/*CLDN4*/*CLDN7* and *NF-YAl*/*NF-YAs* mRNAs. **a** The left heatmap shows: (i) the original classification (first column) of the STAD cell lines available in the CCLE dataset cell lines [30]; (ii) a second classification of the STAD cell lines obtained with the application of the *DeepCC* deep learning tool (second column); (iii) the classification of the STAD cell lines generated following integration with ssGSEA data (third column). To define the Claudin^low^ cell lines we consider a ssGSEA *z-score* value > 1. The right panel shows barplots indicating the TPM (*Transcripts Per Million*) expression levels of the *CLDN3*/*CLDN4*/*CLDN7*, *VIM* (vimentin), *CDH1* (E-Cadherin), *NF-YAl* and *NF-YAs* mRNAs. The rightmost barplot shows the *NF-YAl*/*NF-YAs* expression ratio values. **b** The panel illustrates qRT-PCR determination of *CLDN3*/ *CLDN4*/*CLDN7*/*VIM*/*CDH1*/*NF-YAl*/*NF-YAs* mRNA expression levels in HS746T, LMSU, MKN7, NCIN87, MKN45 and AGS cell lines. The data are expressed as the MEAN ± SD of the values determined. **c.** Western Blot analysis showing the amounts of the *NF-YAl*, *NF-YAs*, *VIM* and *CDH1* proteins in the HS746T, LMSU, MKN7, NCIN87, MKN45 and AGS cell lines

A first classification of the cell lines, based on the ACRG subtypes, was provided by Lee et al. [30]. However, as illustrated in Fig. 7a, this classification leaves 21 cell lines as unclassified, and it does not include the Claudin^low^ subtype. To obtain a more inclusive classification, we employ the *DeepCC* deep-learning tool [36] with the Lee et al. categories serving as the training set. Thereafter, we use the ssGSEA (single-sample Gene Set Enrichment Analysis) platform and our 158-gene signature to identify the Claudin^low^ subgroup, following the ranking of the cell lines according to their *z-score* values. With the exception of 3 cell lines (RERF-GC1B, NUGC3, and TGBC11TKB), all other lines are classified in specific ACRG subtypes (Fig. 7a). Given the >1 ssGSEA *z-score values,* 9 STAD cell lines classify as Claudin^low^ (Supplementary Table 5). With the exception of NUGC2 (MSS/TP53^−^) and RERF-GC1B (unclassified), these Claudin^low^ cell lines derive from the EMT subgroup gathered from the *DeepCC* analyses.

In parallel, we evaluated the expression levels of the mRNAs encoding *CLDN3*/*CLDN4*/*CLDN7*, the EMT marker *VIM* (Vimentin), the epithelial marker *CDH1* (Cadherin-1) and the *NF-YAl*/*NF-YAs* (Fig. 7a). As expected, the Claudin^low^ cell lines are characterized by low/undetectable levels of the 3 claudins. The only exception is represented by the NUGC2, HS746T and RERF-GC1B cell lines, which express significant amounts of the *CLDN4* transcript. The EMT and MSI cell lines characterized by positive *z-score* values show low *CLDN3*, *CLDN4* and *CLDN7* levels. By converse, the MSS/TP53^−^ and MSS/TP53^+^ cell lines, with the exception of FU97, present with negative *z-score* values and high levels of the 3 claudins. The *VIM* mRNA is expressed in 7 of the 9 Claudin^low^, 2 of the 5 EMT and in 1 of the 5 MSI cell lines. The *CDH1* transcript is expressed in 2 Claudin^low^ cell lines presenting with undetectable *VIM* mRNA levels (NUGC2/RERFGC-1B), one EMT (MKN7) and one MSI (NCC59) cell line (Fig. 7a). As expected, the majority of the cell lines characterized by an “epithelial” phenotype express large amount of *CDH1* mRNA. Finally, the expression of *NF-YAl* and *NF-YAs* is associated with two separate “high-to-low” and “low-to-high” TPM *(Transcripts per Millions)* gradients in all the STAD cell lines, with the exception of the MSI SNU1 cell line.

We validated the RNA-seq data by performing qRT-PCR studies to measure the amounts of *CLDN3*, *CLDN4*, *CLDN7*, *VIM*, *CDH1*, *NF-YAl*, and *NF-YAs* in several STAD cell lines (Fig. 7b). With the exception of *CLDN4* in the HS746T cell line, the qRT-PCR data indicate that the selected Claudin^low^ cell lines lack *CLDN3*, *CLDN4*, and *CLDN7* expression. In line with their epithelial characteristics, the MSS/TP53^−^NCIN87 and MKN45 cell lines express *CLDN3*, *CLDN4*, and *CLDN7*, while the MSS/TP53^+^ AGS cell line expresses only *CLDN4* and *CLDN7*. Surprisingly, high *CLDN3*, *CLDN4*, and *CLDN7* expression levels are also observed in the EMT MKN7 cell line. As for *VIM*, *CDH1*, *NF-YAl*, and *NF-YAs*, the qRT-PCR and the RNA-seq data are entirely consistent. Overall, the qRT-PCR results confirm the RNA-seq analyses, with a single inconsistency represented by *CLDN3* expression in the MKN7 cell line.

The intra-cellular expression of a specific mRNA does not necessarily translate into the production of the encoded polypeptide. We address this point in the case of *NF-YAl*, *NF-YAs*, *VIM*, and *CDH1* transcripts, as the presence/absence of the corresponding protein may be of functional importance for the homeostasis of the neoplastic cell. Thus, we determined the levels of the *NF-YAl*, *NF-YAs*, *VIM*, and *CDH1* proteins in the STAD cell lines used for the qRT-PCR validation experiments (Fig. 7c). The results obtained indicate that the amounts of the *NF-YAl*, *NF-YAs*, *VIM*, and *CDH1* proteins determined in each cell line reflect the expression levels of the corresponding transcripts in our qRT-PCR. Specifically, both the qRT-PCR and RNA-Seq data indicate that *CDH1* is absent in the AGS cell line, despite its “epithelial” classification. On the other hand, we note that the only cell line classified as EMT expressing *CDH1* is the MKN7 that we use here. We conclude that Western Blot Analysis is in line with the qRT-PCR shown above.

## Discussion

Our work confirms the existence of a Claudin^low^ subgroup of gastric cancers and provides a better definition of this tumor type. In a previous report [29], we used the collective expression of *CLDN3*, *CLDN4*, and *CLDN7* to identify signature genes capable of identifying specific subcategories of epithelial cancers, with particular reference to STAD.

In BRCA, the notion that Claudin^low^ tumors represent a distinct subgroup was challenged, as some authors proposed to consider the low levels of Claudins as a secondary feature of “classic” BRCA subtypes [22]. In BRCA IHC studies, the data gathered with the use of Claudins as tumor markers posed problems in terms of consistency, coordinated expression, and staining of the proteins located inside or outside the cell [13]. This prompted us to evaluate *CLDN3*, *CLDN4*, and *CLDN7* expression in the 79 STAD tumors of the TCGA database that we previously classified as Claudin^low^ according to the Nishijima signature. The expression differences of the *CLDN3*, *CLDN4*, and *CLDN7* genes between the Claudin^low^ and the other STAD subtypes were minor and statistically insignificant (Fig. S1).

For this reason, a more precise definition of the Claudin^low^ STADs was necessary. This is now obtained with the use of our 158-gene signature common to STAD and BRCA tumors [23]. Application of this signature in hierarchical clustering of the TCGA samples captures a set of 56 Claudin^low^ cancers, which originates from the EMT subset of the ACRG stomach tumors. Relative to the other subgroups, including EMT, the identified Claudin^low^ set does show statistically reduced levels of *CLDN3*, *CLDN4*, and *CLDN7*.

In addition, our 158-gene signature identifies the subset in three databases of 231, 60, and 13 STAD tumors.

While gene expression differences between the “epithelial” subtypes, Claudin^low^ and EMT are quite substantial, they are more subtle between these two latter subtypes. Yet, statistically robust terms emerged in GO and Reactome pathways analysis comparing EMT and Claudin^low^. Notably, cell adhesion prevails in Claudin^low^, and proliferative terms, particularly linked to mitosis, in EMT: this could suggest a further acquisition of migratory properties of the formers.

We used the AI-based *DeepCC* tool and the 158-gene signature to obtain a classification of the 50 STAD cell lines present in CCLE into one of the 5 subgroups identified with the approaches discussed above. The results were validated by wet experiments performed on 6 selected cell lines. Overall, our data confirm a previous classification based on 29 cell lines [30], with the sole exception of the SNU719 cell line, which we classify as MSS/TP53^+^ instead of EBV. In addition, our results provide a specific classification of 19 of the previously unclassified 21 cell lines. Finally, they permit the identification of a group of 9 Claudin^low^ cell lines previously classified as EMT. It is worth mentioning that while the expression of *CLDN4* and *CLDN7* are generally concordant, the expression of *CLDN3* is not always coherent in the TCGA (Fig. 1c), PRJEB43867 (Fig. S3 sample GC15), and CCLE STAD cell lines (Fig. 7). This notion could be considered in future IHC studies of STAD samples.

In conclusion, our data support the presence of an aggressive subset of STAD tumors, which can be correctly denominated as Claudin^low^ on the basis of *CLDN3*, *CLDN4*, and *CLDN7* expression. The Claudin^low^ and the EMT subgroups of STADs are separated from each other. It remains to be established whether the 158-gene signature represents an equally useful tool for the definition of the controversial Claudin^low^ subtype in BRCA and in BLCA tumors. With respect to this, it should be emphasized that the fraction of Claudin^low^ tumors is more numerous in the STAD (12/13%) than the BRCA cases of the TCGA dataset (3%, data not shown), which suggests a greater relevance of this phenotype in the former than the latter cancer type.

## Supplementary Information

Below is the link to the electronic supplementary material.

Supplementary file1 (XLSX 9 KB)

Supplementary file2 (XLSX 11 KB)

Supplementary file3 (XLSX 17 KB)

Supplementary file4 (XLSX 644 KB)

Supplementary file5 (XLSX 21 KB)

Supplementary file6 (PNG 994 KB) Fig S1. *CLDN3*, *CLDN4*, *CLDN7*, *NFYAl* and *NFYAs* expression in STAD tumors. Box plots showing the *CLDN3*, *CLDN4*, *CLDN7*, *NFYAl* and *NFYAs* expression levels, as well as the *NFYAl/NFYAs* expression ratio values of the STAD tumors available in the TCGA dataset. The values are shown in CCLE (*Transcripts Per Million*). Tumor types are defined according to the molecular classification proposed by Gallo et al [26]. The p-values are calculated using the Wilcoxon rank-sum test

Supplementary file7 (PNG 4082 KB) Fig S2. Differential Gene Expression between Claudin^low^ and EMT subtypes of STAD tumors. a. Principal Component Analysis (PCA) of transcriptome profiles showing separation among Claudin^low^, EMT, MSI, MSS:TP53⁻, MSS:TP53⁺, and normal adjacent gastric tissue samples. b. Volcano plot displaying differentially expressed genes between Claudin^low^ and EMT subtypes. Significantly upregulated (right) or downregulated (left) genes in Claudin^low^ are shown in red, while non-significant genes are in black. Dashed vertical lines indicate the filtering thresholds: |log₂FC| > 1 and -log10FC > 2. c. Functional enrichment analysis of differentially expressed genes depicted in b. Gene Ontology (left) and Reactome Pathways (right) terms are shown for genes upregulated (top panels, green) and downregulated (bottom panels, red) in Claudin^low^ compared with EMT tumors

Supplementary file8 (PNG 2659 KB) Fig S3. Expression of the *CLDN3/CLDN4/CLDN7* and *NF-YAl/NF-YAs* mRNAs in an Italian cohort of STAD patients. a. The leftmost barplot illustrates the correlation level of the 158-gene signature in the tumor tissues of the13 Italian gastric-cancer (GC) patients belonging to the PRJEB43867 cohort. The correlation level is measured using the median z-score values of the signature genes. The other 3 barplots show the *NF-YAl* and *NF-YAs* expression levels as well as the *NF-YAl/NF-YAs* expression ratio values. The *NF-YAl* and *NF-YAs* values are shown in CCLE. b. The Panel shows the *CLDN3/CLDN4/CLDN7* mRNA expression levels in the tumor tissues. GC samples are classified as *G-DIFF* and *G-INT*, as originally reported [26]
