# Supplementary figures and images for: A gene-expression signature defines a subtype of Stomach Adenocarcinomas with low levels of Claudins and a high ratio of NF-YA long/NF-YA short splicing variants
Source: Gastric Cancer. 2025 Oct 13;29(1):132–46. doi: 10.1007/s10120-025-01671-1 (PMC12830421; doi:10.1007/s10120-025-01671-1)

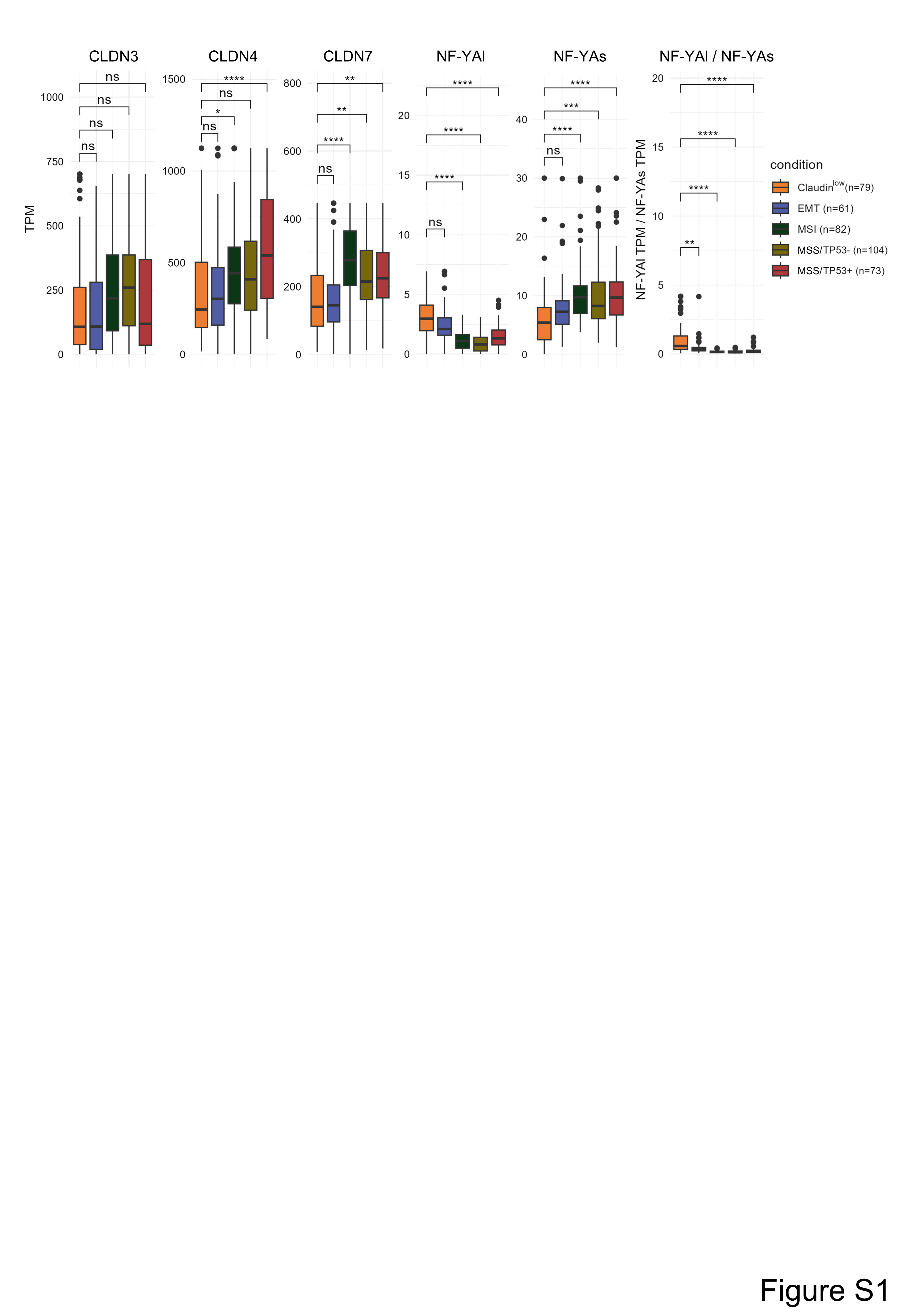

Supplement: Supplementary file 6 — Supplementary file6 (PNG 994 KB) Fig S1. CLDN3, CLDN4, CLDN7, NFYAl and NFYAs expression in STAD tumors. Box plots showing the CLDN3, CLDN4, CLDN7, NFYAl and NFYAs expression levels, as well as the NFYAl/NFYAs expression ratio values of the STAD tumors available in the TCGA dataset. The values are shown in CCLE (Transcripts Per Million). Tumor types are defined according to the molecular classification proposed by Gallo et al [26]. The p-values are calculated using the Wilcoxon rank-sum test [file 10120_2025_1671_MOESM6_ESM.png]

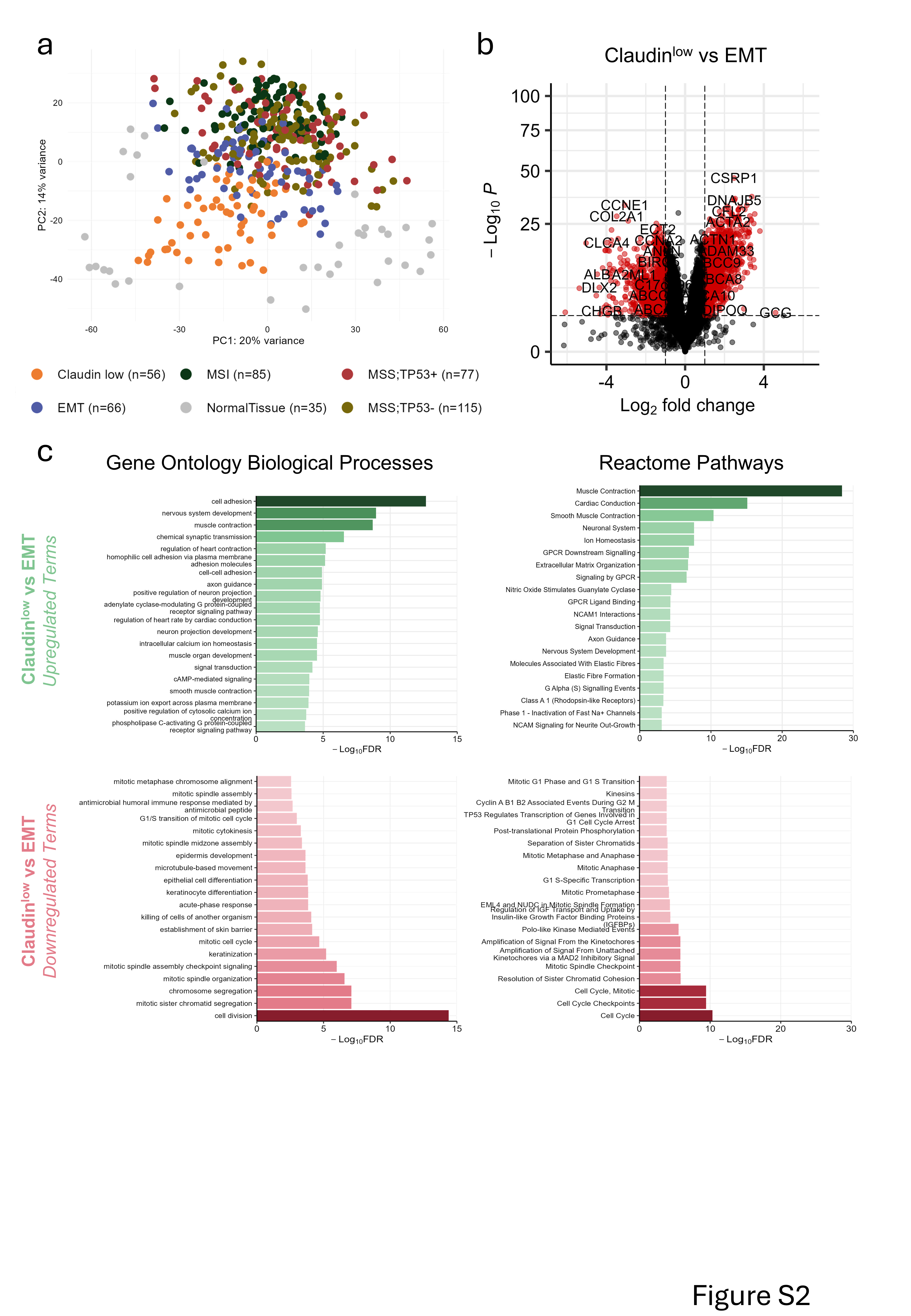

Supplement: Supplementary file 7 — Supplementary file7 (PNG 4082 KB) Fig S2. Differential Gene Expression between Claudinlow and EMT subtypes of STAD tumors. a. Principal Component Analysis (PCA) of transcriptome profiles showing separation among Claudinlow, EMT, MSI, MSS:TP53⁻, MSS:TP53⁺, and normal adjacent gastric tissue samples. b. Volcano plot displaying differentially expressed genes between Claudinlow and EMT subtypes. Significantly upregulated (right) or downregulated (left) genes in Claudinlow are shown in red, while non-significant genes are in black. Dashed vertical lines indicate the filtering thresholds: |log₂FC| > 1 and -log10FC > 2. c. Functional enrichment analysis of differentially expressed genes depicted in b. Gene Ontology (left) and Reactome Pathways (right) terms are shown for genes upregulated (top panels, green) and downregulated (bottom panels, red) in Claudinlow compared with EMT tumors [file 10120_2025_1671_MOESM7_ESM.png]

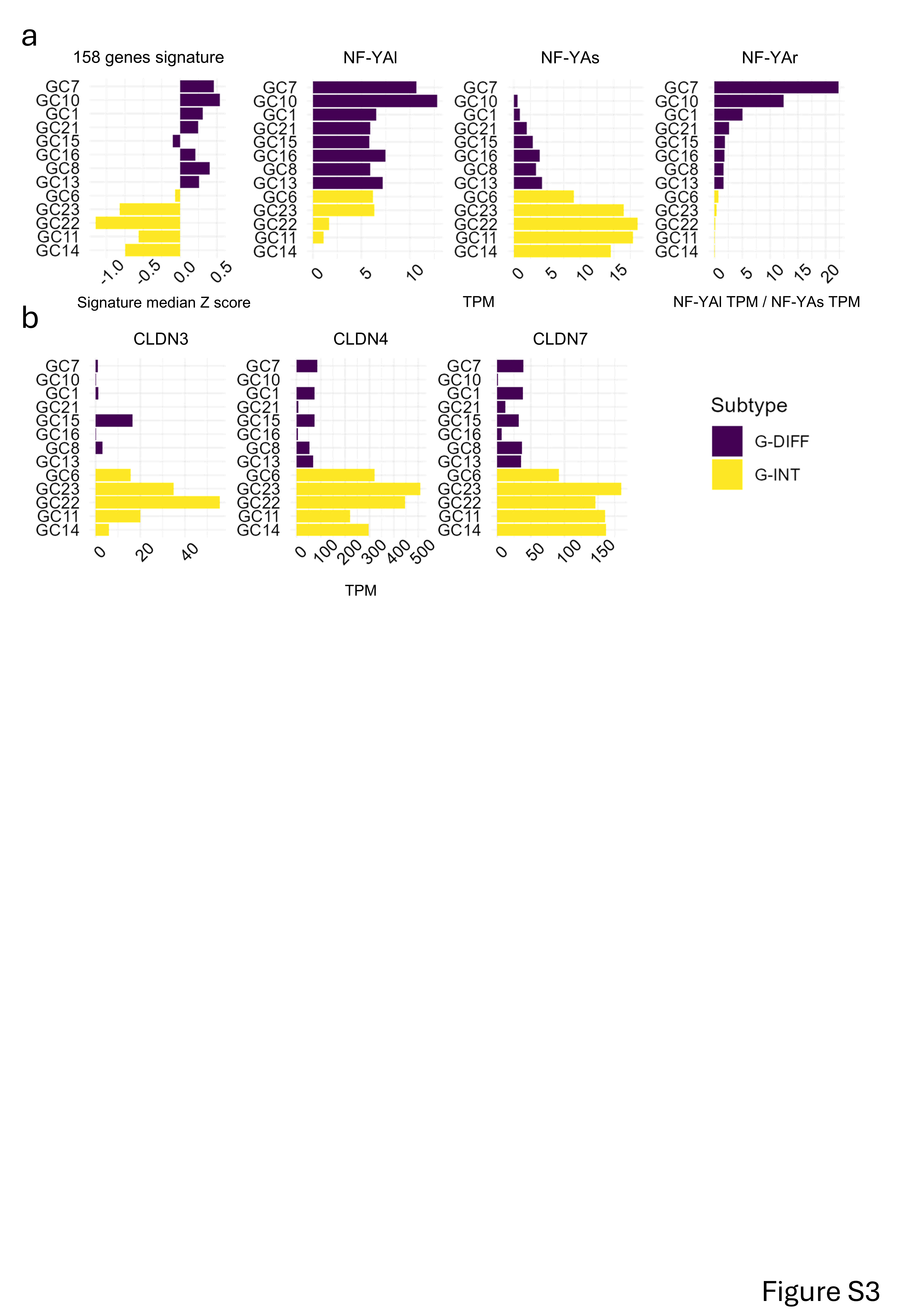

Supplement: Supplementary file 8 — Supplementary file8 (PNG 2659 KB) Fig S3. Expression of the CLDN3/CLDN4/CLDN7 and NF-YAl/NF-YAs mRNAs in an Italian cohort of STAD patients. a. The leftmost barplot illustrates the correlation level of the 158-gene signature in the tumor tissues of the13 Italian gastric-cancer (GC) patients belonging to the PRJEB43867 cohort. The correlation level is measured using the median z-score values of the signature genes. The other 3 barplots show the NF-YAl and NF-YAs expression levels as well as the NF-YAl/NF-YAs expression ratio values. The NF-YAl and NF-YAs values are shown in CCLE. b. The Panel shows the CLDN3/CLDN4/CLDN7 mRNA expression levels in the tumor tissues. GC samples are classified as G-DIFF and G-INT, as originally reported [26] [file 10120_2025_1671_MOESM8_ESM.png]
